# 2441. Implementation of Decolonization Strategies and Rates of Bacteremia in ECMO Patients

**DOI:** 10.1093/ofid/ofad500.2060

**Published:** 2023-11-27

**Authors:** Madyson Taylor, Rachael A Lee, Russell Griffin, Catina James, Angela Akinsanya, Mary duncan, Jeremey Walker

**Affiliations:** University of Alabama at Birmingham, Birmingham, Alabama; University of Alabama at Birmingham, Birmingham, Alabama; University of Alabama at Birmingham, Birmingham, Alabama; University of Alabama - Birmingham, Birmingham, Alabama; University of Alabama at Birmingham, Birmingham, Alabama; University of Alabama-Birmingham, birmingham, Alabama; University of Alabama Birmingham, Birmingham, Alabama

## Abstract

**Background:**

To date, there is little data on decolonization in extracorporeal membrane oxygenation (ECMO) patients and if decolonization efforts improve rates of bacteremia with multi-drug resistant organisms (MDROs). We sought to determine if implementation of universal nasal decolonization with daily chlorhexidine bathing will decrease blood stream infections (BSI) in patients undergoing ECMO.

**Methods:**

A retrospective cohort study was conducted of all patients with admitted to UAB Hospital and placed on ECMO from 12/22/2017-6/1/2022. Data collected included baseline patient characteristics and clinical data, microbiology data, interventions for source control, dates of line placement, appropriate antibiotic treatment, and outcomes. Categorical variables were analyzed with the Fisher exact test, and continuous variables analyzed with the t test or Wilcoxon rank-sum test when appropriate. Only variables found to be statistically significant will be included in the model. A P value < .05 are considered significant.

**Table 1.**
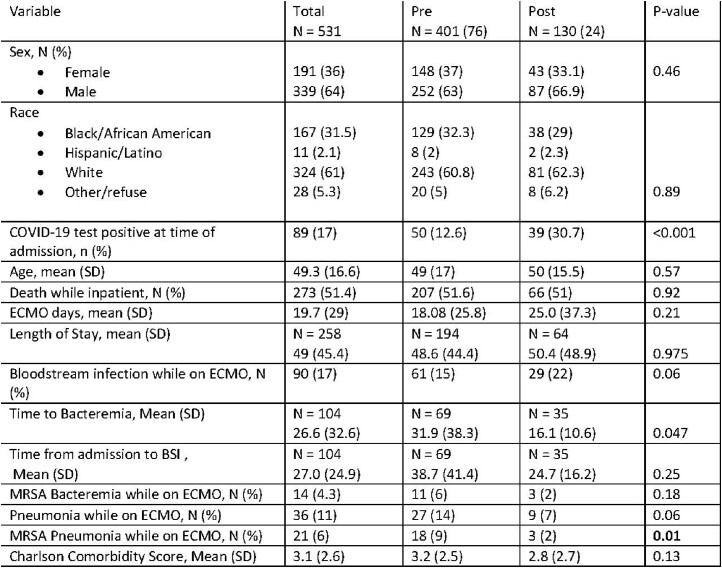
Group Demographics

**Results:**

531 patients placed on ECMO were included in this study and separated into groups: Pre implementation: 401 (76%), post implementation: 130 (24%). (Table 1). 61 (15%) pre patients developed bacteremia with an average of 18.1 ECMO days. 29 (22%) of post patients had a BSI with an average of 25.0 ECMO days. While the average number of BSI per 100 ECMO days increased in the post-implementation group (0.58/100 ECMO days to 1.02/100 ECMO days), the overall incidence rate of MRSA decreased (0.22/100 ECMO days vs 0.09/100 ECMO days). Of note, 89 (17%) patients were found to have COVID-19 at time of ECMO, 12.6% of the pre-implementation group vs 31% of the post-implementation group, (p < 0.001).

Incidence Rate: Number of BSI per ECMO Days

**Figure:**
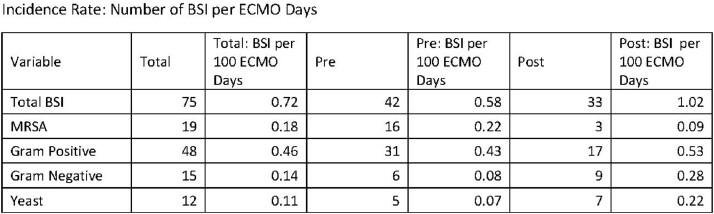

**Conclusion:**

There was no statistical significance between the pre and post groups in the study with the amount of time on ECMO, the time from ECMO cannulation to BSI, the amount of BSIs or the amount of MRSA bacteremia. Implementation of universal decolonization with daily CHG reduced rates of MRSA bacteremia. The lack of research in this field and the timing of this study almost exclusively assessing ECMO patients during the COVID-19 pandemic during the post-implementation period warrants further investigation on if decolonization strategies can reduce BSIs and MDROs bacteremia in ECMO patients.

**Disclosures:**

**All Authors**: No reported disclosures

